# Systematic development and implementation of interventions to OPtimise Health Literacy and Access (Ophelia)

**DOI:** 10.1186/s12889-017-4147-5

**Published:** 2017-03-03

**Authors:** Alison Beauchamp, Roy W. Batterham, Sarity Dodson, Brad Astbury, Gerald R. Elsworth, Crystal McPhee, Jeanine Jacobson, Rachelle Buchbinder, Richard H. Osborne

**Affiliations:** 1 Health Systems Improvement Unit, Centre for Population Health, Deakin University, Geelong, VIC Australia; 2 Department of Epidemiology and Preventive Medicine, Monash University, Melbourne, Australia; 3 Fred Hollows Foundation, Carlton, VIC Australia; 4 Melbourne Graduate School of Education, University of Melbourne, Parkville, VIC Australia; 5 Victorian Department of Health and Human Services, Melbourne, Australia; 6 Monash Department of Clinical Epidemiology, Cabrini Hospital, Malvern, VIC Australia

**Keywords:** Health literacy, Health inequities, Ophelia, Chronic disease, Health Literacy Questionnaire, HLQ, Health service improvement, Healthcare access

## Abstract

**Background:**

The need for healthcare strengthening to enhance equity is critical, requiring systematic approaches that focus on those experiencing lesser access and outcomes. This project developed and tested the Ophelia (OPtimising HEalth LIteracy and Access) approach for co-design of interventions to improve health literacy and equity of access. Eight principles guided this development: Outcomes focused; Equity driven, Needs diagnosis, Co-design, Driven by local wisdom, Sustainable, Responsive and Systematically applied. We report the application of the Ophelia process where proof-of-concept was defined as successful application of the principles.

**Methods:**

Nine sites were briefed on the aims of the project around health literacy, co-design and quality improvement. The sites were rural/metropolitan, small/large hospitals, community health centres or municipalities. Each site identified their own priorities for improvement; collected health literacy data using the Health Literacy Questionnaire (HLQ) within the identified priority groups; engaged staff in co-design workshops to generate ideas for improvement; developed program-logic models; and implemented their projects using Plan-Do-Study-Act (PDSA) cycles. Evaluation included assessment of impacts on organisations, practitioners and service users, and whether the principles were applied.

**Results:**

Sites undertook co-design workshops involving discussion of service user needs informed by HLQ (*n* = 813) and interview data. Sites generated between 21 and 78 intervention ideas and then planned their selected interventions through program-logic models. Sites successfully implemented interventions and refined them progressively with PDSA cycles. Interventions generally involved one of four pathways: development of clinician skills and resources for health literacy, engagement of community volunteers to disseminate health promotion messages, direct impact on consumers’ health literacy, and redesign of existing services. Evidence of application of the principles was found in all sites.

**Conclusions:**

The Ophelia approach guided identification of health literacy issues at each participating site and the development and implementation of locally appropriate solutions. The eight principles provided a framework that allowed flexible application of the Ophelia approach and generation of a diverse set of interventions. Changes were observed at organisational, staff, and community member levels. The Ophelia approach can be used to generate health service improvements that enhance health outcomes and address inequity of access to healthcare.

**Electronic supplementary material:**

The online version of this article (doi:10.1186/s12889-017-4147-5) contains supplementary material, which is available to authorized users.

## Background

The recent transition from the Millennium to the Sustainable Development Goals has led to a renewed global focus on health and equity [1, 2]. Yet despite increased spending on healthcare, the burden of non-communicable disease continues to grow [3] and socioeconomic gradients in health continue to widen [4, 5]. Appropriate responses will require new systematic approaches that address persisting inequalities and are built upon detailed knowledge of local populations. Interventions developed in one population or setting may not be relevant in other settings, and it may be difficult to embed interventions within a service if they do not fit the needs of the population group or take local contexts into account [6, 7].

Health literacy is a multi-dimensional concept, described by the World Health Organisation as “the cognitive and social skills which determine the motivation and ability of individuals to gain access to, understand and use information in ways which promote and maintain good health” [8]. Information about the health literacy of people in a community can offer health and community organisations insight into the challenges people experience when trying to access and engage with their services.

The Health Literacy Questionnaire (HLQ) is a measure of health literacy designed to capture and measure all aspects of the concept. The HLQ comprises nine separate scales, each describing a different aspect of health literacy. People’s scores on each scale reflect both their personal health literacy abilities and the experiences they have had as they attempt to engage with health information and health services [9]. The nine scales are: 1) Feeling understood and supported by healthcare providers; 2) Having sufficient information to manage my health; 3) Actively managing my health; 4) Social support for health; 5) Appraisal of health information; 6) Ability to actively engage with healthcare providers; 7) Navigating the healthcare system; 8) Ability to find good health information; and 9) Understand health information well enough to know what to do. In combination, these scales provide a profile of a person’s health literacy strengths and needs. The HLQ has been shown to have strong measurement properties in a number of different settings [9–12].

**Fig. 1:**
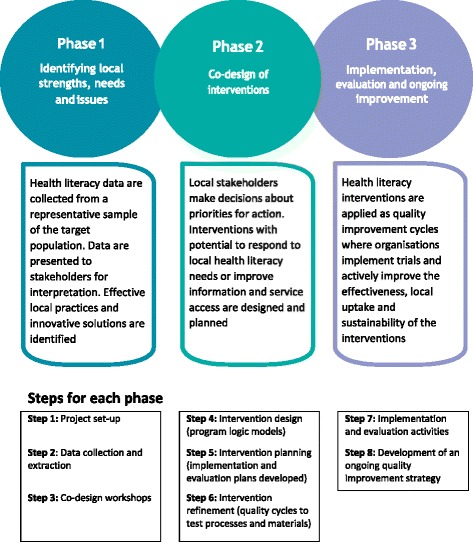
Phases of the Ophelia approach

Scores on the nine scales are not combined, rather profiles showing the areas of strength and needs across the nine scales are produced. The HLQ thus provides a mapping of health literacy needs for individuals and groups of individuals. This, in turn, informs an approach to health system strengthening through a) optimising the health literacy of individuals and, b) optimising the health literacy responsiveness of organisations. This approach, called Ophelia (OPtimising HEalth LIterAcy and Access) [7] includes three key phases as shown in Fig. 1. Phase 1 involves undertaking a health literacy needs assessment on a representative cross-section of people associated with a service or sector. The results of this assessment are then presented to stakeholders for discussion and generation of ideas for service or practice strengthening. In Phase 2, a program logic model is constructed, and processes and resources to support implementation of interventions are developed and refined using quality improvement cycles. In Phase 3, continuous quality improvement processes are applied to implement, refine and evaluate the intervention.

The theoretical underpinnings of the Ophelia approach are described in the protocol for a large multi-centred partnership project conducted in Victoria, Australia (hereafter called Ophelia Victoria) [7]. The partnership was co-designed by academic teams from two Universities, three sections within the state government Department of Health and Human Services and nine health service sites across Victoria.

The overall aim of Ophelia Victoria was to develop and test a structured approach that organisations can use to enhance equitable engagement of consumers in health and health care. There was considerable variation in both the context in which development and testing occurred (i.e., type of partnering organisation) and in the nature of the potential interventions (i.e., from health promotion, to clinical services, to organisational policy). This variability required that testing of the Ophelia approach be applied with considerable flexibility. As such, a set of predefined principles were established to guide the project’s operationalisation (Table 1). In this paper, we report the application of the Ophelia process in a proof-of-concept study. Proof-of-concept was defined as successful application of the eight underlying principles to achieve the development of health literacy-informed interventions with the potential to impact on health and equity outcomes.

**Table 1:** The Ophelia (OPtimising HEalth LIteracy and Access) principles that guide the aims, development and implementation of structured interventions to improve health and equity outcomes in communities

1. Outcomes focused	• Improved health and reduced health inequities
2. Equity driven	• All activities at all stages prioritise disadvantaged groups and those experiencing inequity in access and outcome
3. Co-design approach	• In all activities at all stages, relevant stakeholders engage collaboratively to design solutions
4. Needs-diagnostic approach	• Participatory assessment of local needs using local data
5. Driven by local wisdom	• Intervention development and implementation is grounded in local experience and expertise
6. Sustainable	• Optimal health literacy practice becomes normal practice and policy
7. Responsiveness	• Recognise that health literacy needs and the appropriate responses vary across individuals, contexts, countries, cultures and time
8. Systematically applied	• A multilevel approach in which resources, interventions, research and policy are organised to optimise health literacy

## Methods

### Participating sites

Expressions of interest to participate in the project were sought from service organisations providing Home and Community Care (HACC) services, Hospital Admission Risk Programs (HARP) or community nursing and other chronic disease services [7]. Eight organisations were recruited with one large organisation subsequently separating into two program areas, resulting in nine participating sites. Details of participating organisations are shown in Table 2, including a brief description of services relevant to this project. Each site was required to establish their own project team to lead activities at their site and to liaise with the academic research team. Approval to conduct the study was obtained from three of the participating sites with their own ethics committees, while the remaining six sites accepted approval from the Deakin University Human Research Ethics Committee. Written informed consent was obtained from all participants for all phases of the study, including clients and practitioners.

**Table 2:** Description of each participating service organisation

Site ID. Service type	Relevant services delivered	Initial project aims/target group for needs assessment
Site #1 Metropolitan municipal council	Ongoing home and community care services (aged care support for activities of daily living, planned activity groups)	To support clients to improve social connectedness, physical/mobility and remain connected to their community while living at home. *Target group:* Clients aged 65+ with mobility issues who are difficult to motivate and engage
Site #2 Metropolitan municipal council	Ongoing home and community care services (aged care support for activities of daily living, delivered meals)	Understand how vulnerable clients find, understand and use health information. Specifically to identify indicators for assessment officers to improve their capacity to identify clients with health literacy needs *Target group:* All delivered meals clients (a vulnerable client group, i.e. frail aged and socially isolated)
Site #3 Metropolitan community nursing service	Ongoing care for clients at home with chronic conditions including education of clients in self-management	Improved awareness and uptake among all nurses of a standardised approach to diabetes education so that clients are better able to independently self-manage their condition. *Target group:* Clients with diabetes and long term wounds
Site #4 Rural community health service	Ongoing and episodic care for clients and community members (on-site and outreach allied health, outreach health promotion including community activity groups)	Engage people from rural and remote communities with (or at risk of) chronic disease to better manage their health, navigate the health system and develop effective relationships with health professionals. *Target group:* All clients eligible for services including those with complex conditions
Site #5 Rural community health service	Ongoing and episodic care for clients and community members (on-site allied health, outreach health promotion including community activity groups)	To support clients living with chronic disease and who are disadvantaged to access services and programs. Goals are that clients will: 1) be aware of what health professionals are involved in their care; 2) be aware of how to access health services to assist with chronic disease self-management. *Target group:* All clients with chronic disease eligible to receive our services
Site #6 Metropolitan community health service	Ongoing and episodic care for clients and community members (on-site allied health services, community- and centre-based activity groups)	To tailor services to meet the different health literacy needs of clients and to improve clients’ skills and capacity to access services *Target group:* Clients with chronic and complex conditions attending planned activity groups and those who receive 1:1 clinical interventions
Site #7 Outer metropolitan community health service	Ongoing and episodic care for clients and community members (on-site allied health services, community nursing service)	To increase community awareness of, and engagement with the service to help people develop the skills to self-manage their health conditions. *Target group:* Community members not currently engaged; Existing clients who do not achieve ongoing engagement; Clients with chronic conditions who would benefit from improved self-management skills
Site #8 Metropolitan hospital Admission Risk Program	Ongoing (6–12 weeks) intensive case management for clients with chronic disease at risk of hospital admission. Allied health, nursing	To evaluate and improve the service’s response to patients who are in a crisis (defined by patient) to determine if the response is flexible, appropriate and timely resulting in reduced unplanned readmissions. *Target group:* All current clients of the service
Site #9 Regional metropolitan Hospital Admission Risk Program	Ongoing (6–12 weeks) intensive case management for clients with chronic disease at risk of hospital admission. Allied health, primarily nursing	To improve clients’ capacity to access and understand health information and attend appointments. To provide clinicians with a consistent framework for approaching care *Target group:* All current clients of the service

## Measurement of health literacy

The Health Literacy Questionnaire (HLQ) was designed using a grounded, validity-driven approach and initially tested in diverse samples of individuals in Australian communities where it was shown to have strong construct validity, reliability and acceptability to clients and clinicians [9, 13]. It can be self-administrated or administered in an interview, ensuring inclusion of people who cannot read or have other difficulties with self-administration. The HLQ contains 44 questions across nine separate scales (see background). Response options for each scale were determined by the content and nature of the items. For scales 1–5 four-point ordinal response options are used (Strongly Disagree, Disagree, Agree and Strongly Agree), while for scales 6–9 five-point ordinal response options are used (Cannot Do, Very Difficult, Quite Difficult, Quite Easy and Very Easy). The psychometric properties of the HLQ were tested in the current study sample [13]. All HLQ scales were found to have strong construct validity, be homogenous, and with good to excellent composite reliability ranging from 0.80 to 0.89. With a small number of exceptions, strict measurement invariance was seen across the participating organisations and the gender, language, age and educational level of respondents [13].

### Application of the Ophelia process

In Phase 1 of the Ophelia process, each site was asked to define a broad aim for their project and identify a priority group where health literacy was thought to contribute to inequitable service access or poor health outcomes. Staff at each site then undertook a needs assessment of a representative sample of clients in their target group by collecting HLQ and demographic data [10]. The researchers also conducted semi-structured interviews with up to three participants, randomly sampled from within tertiles of HLQ scale scores. The interviews explored the thinking underlying participants’ responses to the HLQ (see Additional file 1: Phase one interviews for semi-structured interview template). Participants with higher and lower scale scores were selected in order to capture individual health literacy strengths and areas of need. Data from these interviews were used to support interpretation of the HLQ and to provide context and narrative for case studies that were used in presenting the data.

Cluster analysis of HLQ scores, within each site, was used to identify subgroups of people with health literacy profiles that were similar within each subgroup, yet distinct from other subgroups (see below for specific statistical procedure). Short vignettes (narratives) were written to represent a typical person within each subgroup. Three-hour workshops were held at each site with healthcare practitioners and managers who were familiar with the target group, the service culture, and the context within which each service operated. Each workshop was facilitated by a member of the research team and observed by others for training purposes. A co-design approach was used, whereby participants collectively raised and discussed ideas about strategies that they currently use, or could use, to support the persons described in the narratives. Workshop participants were encouraged to consider solutions at the individual client-level first, and then solutions at the organisation level. The ideas generated from these workshops provided a pool of potential solutions to the priority health issues identified at the start of the project.

In Phase 2, based on their overall project aims and the solution ideas generated in the workshops, sites were supported to develop a program logic model to describe the service improvements they wished to undertake. The program logic model presented the elements of the intervention and aligned these with desired changes to their target group’s health literacy. A rapid literature review for evidence to support the selected interventions was also conducted. Based on their program logic model and relevant evidence, sites then developed detailed implementation and evaluation plans. The template for these plans is shown as additional data (see ‘Additional file 2: Implementation plan template’). A workshop was held at this point, facilitated by the research team, in which project team members from the nine participating sites came together to share and refine their intervention ideas. Over 2–3 months, Plan Do Study Act (PDSA) quality improvement cycles were used to develop and refine materials and processes in preparation for implementation of the selected interventions. A second workshop was held so that organisations could discuss, compare and further refine their findings from this pre-testing and development phase.

In Phase 3, interventions were implemented over a period of up to 6 months, with evaluation of the interventions occurring in accordance with the Ophelia protocol [7]. Evaluation of each intervention involved collection of qualitative and quantitative data using convenience sampling in most cases. For quantitative data, five sites administered between two to three HLQ scales to intervention participants before and after delivery of the intervention. Several of these sites also selected one HLQ scale in which they hypothesised no change would occur (comparison scale). One site also administered a diabetes knowledge questionnaire to participants before and after the intervention [14]. Other quantitative data included rates of participation and uptake of the intervention where relevant. Qualitative data collection included focus groups with practitioners who were involved in delivery of interventions, and semi-structured interviews with convenience samples of participating clients and volunteers. The interviews sought to uncover the impact of the interventions on individuals and their health literacy and/or other mechanisms by which the intervention might achieve impacts (see Additional file 3 for templates for client, volunteer and practitioner interviews). Case studies of individual clients were also collected from two sites for evaluation purposes. Phase 3 of the Ophelia process also sought to embed interventions into existing organisational processes and service delivery.

### Data analysis

Data were analysed using SPSS Version 22 [15] and Stata Version 13 [16]. In Phase 1, hierarchical cluster analysis was undertaken using Ward’s method for linkage as previously described [7]. Cluster analysis data were presented as means (SD) for each of the scale scores in each of the clusters. In Phase 3, the magnitude of pre-post change in HLQ scales was assessed using Cohen’s d effect size with 95% confidence intervals [17]. Participation and uptake rates were presented as numbers and percentages. For interview findings, data were thematically analysed using NVivo 10 [18]. The interview questions were utilised as starting points and data from the transcripts were coded into these initial themes. These initial themes were represented as “parent nodes" within NVivo. Once all relevant data from the transcripts were coded into the parent nodes, “child nodes” were created to represent any sub themes that emerged during further analysis. The sequence of coding followed the general structure outlined by Saldaña [19] and while a number of the coding methods that he described were used, the dominant methods were process and causal coding. Demographic data for Phases 1 and 3 were presented as means and SD for continuous data and proportions for categorical data.

## Results

### Phase 1

The target groups and project aims selected by each of the participating organisations are shown in Table 3. In line with the type of client routinely seen by the organisations, most target groups included older clients with chronic conditions. Project aims for each site focused on improving clients’ health through mechanisms such as enhancing existing client or community members’ ability to self-manage their health, understand health information, or engage more effectively with healthcare providers.

**Fig. 2:**
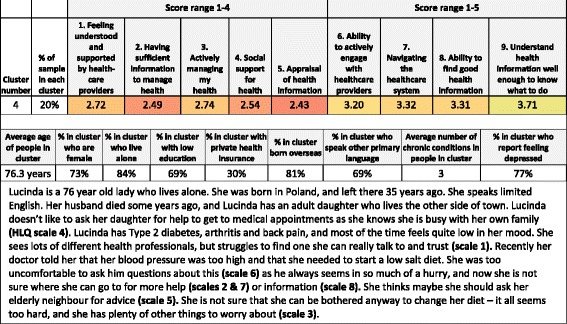
Example of a narrative (vignette) derived from cluster analysis and related health literacy* and demographic data

A detailed description of the overall client sample (*n* = 813) from the needs assessment has been reported elsewhere [10]. Briefly, the mean age of clients was 72.1 (range 19–99) years. Females comprised 63% of the sample, 48% had not completed secondary education, and 96% reported at least one existing health condition. Key findings from each organisation’s needs assessment are shown in Table 3. The needs assessment identified lower health literacy for many clients, with cluster analysis revealing between 8 and 15 subgroups with different health literacy profiles at each site. Between 4 and 6 narratives describing a range of these subgroups were written for each site (*n* = 41 narratives in total). An example of one health literacy profile, demographic data and its narrative is shown in Fig. 2.

**Table 3:** Findings from site-level needs assessment, overview of interventions developed in response to needs assessment, and the focus of each intervention

Needs assessment findings	Intervention aim and overview	Focus of intervention
Site #1 Metropolitan municipal council		
Many clients lacked confidence in their ability to find and appraise health information, and actively manage their health. Many clients also indicated a low level of social support for health, and difficulties engaging with healthcare providers. Some were found to have higher health literacy overall	The intervention aimed to support volunteers with higher health literacy to act as mentors for disadvantaged, frail, older community members during exercise programs run by the municipal council.	Community volunteers act as local health mentors and so build community members’ capacity to achieve better health outcomes
Site #2 Metropolitan municipal council		
Many clients had poor information appraisal skills and found it difficult to engage with healthcare providers. In the workshop staff noted some clients were passive in their interactions with their general practitioner (GP) and were unwilling to discuss their failing health with GPs in case they were put into institutional care.	The intervention aimed to empower clients to optimise their relationship with their GP by screening for client-GP engagement issues and then providing appropriate guidance to clients	Directly improve clients’ health literacy through providing resources or targeted training
Site #3 Metropolitan community nursing service		
Many clients experienced difficulties actively managing their health, and struggled to find and appraise health information. Higher scores were seen for trusting healthcare providers. Staff identified inconsistencies in the way diabetes education was delivered across the service, and reported clients were provided with information from multiple sources, which is often unread.	The intervention aimed to improve the quality of diabetes education within the service by using an education checklist and online library of staff resources, tailoring education to each client’s learning style, and use of the teach-back method.	Target clinicians through provision of skills training and resources to support them to build clients’ capacity to self-manage their long term condition
Site #4 Rural community health service		
Many clients lack sufficient health information, and reported difficulties navigating the health care system and appraising and understanding health information. Many clients had geographical challenges to accessing care and information. The service has an active pool of volunteers, many of whom would like to be more involved.	The intervention aimed to build community capacity to self-manage health by training existing volunteers of the health service to act as health mentors in their local rural community	Community volunteers act as local health mentors and so build community members’ capacity to achieve better health outcomes
Site #5 Rural community health service		
Many clients did not have sufficient information to manage their health, lacked social support for health*,* had limited ability to appraise health information, and were unable to find good health information. The area has transport limitations, restricting people’s ability to access healthcare and leading to increased social isolation.	The intervention aimed to build community capacity to navigate health information on the web by providing training and resources primarily in community settings.	Directly improve clients’ health literacy through providing resources or targeted training
Site #6 Metropolitan community health service		
Many clients had limited ability to navigate the healthcare system or to find and understand health information. Clients reported not knowing the role of the service or how to access it. An opportunity for improving service access between co-located Dental Health and Primary Health Services was identified.	The intervention aimed to build clients’ capacity to navigate local services by implementing a referral pathway between co-located dental (for low-income clients) and primary health care services.	Focus on redesigning existing service procedures to improve access to services
Site #7 Outer metropolitan community health service		
Many clients had low scores for social support for health, having sufficient information, navigating the health system and finding health information. Scores for engagement with healthcare providers were higher. Staff in the workshop highlighted difficulties for clients with chronic disease to keep up-to-date with their knowledge.	The interventions included 1) a patient-held record to help clients manage appointments and monitor health goals, 2) a process for Care Coordination in the District Nursing Team was also developed.	1) Directly improve clients’ health literacy through providing resources; 2) Redesign existing service procedures to improve access to services
Site #8 Metropolitan Hospital Admission Risk Program		
Many clients had low scores for social support for health, having sufficient information, navigating the health system and finding health information. Scores for engagement with healthcare providers were higher. The workshop highlighted a need for the service to evaluate how clients in crisis are managed.	The intervention aimed to support clients with service navigation during crises. Involved’navigation’ plans and use of the teach-back method in client education about managing health crises.	Target clinicians through provision of skills training and resources to support them to build clients’ capacity to self-manage their long term condition.
Site #9 Regional metropolitan Hospital Admission Risk Program		
Many clients lacked information to manage their health and had limited capacity to find, understand and appraise health information. Most clients reported a good relationship with healthcare providers. In the workshop, HARP clinicians reported needing a consistent framework for approaching care, and a need for clients to understand the things they can do to manage their health after discharge from the service.	The intervention aimed to improve the way in which clients’ understand and use their care plans by tailoring education to each client’s learning style, and training clinicians in use of teach-back.	Target clinicians through provision of skills training and resources to support them to build clients’ capacity to self-manage their long term condition.

Co-design workshops at each site were attended by between 6 and 24 participants comprising allied health and nursing practitioners with a range of clinical experience, program managers and team leaders and, in two cases, administrative staff. From these workshops, a total of 315 intervention elements were generated (mean number of ideas for each site 40, range 21 – 78). While some intervention ideas were unique to a single vignette, in many cases ideas spanned more than one vignette. In these cases, the ideas incorporated elements specific to the needs of the person described in that vignette. For example, an intervention idea to use volunteers in delivery of health messages was considered at one site to be a potential idea for three vignettes, but clinicians recognised that the method of delivery would need to vary according to the diversity of needs presented in the vignettes.

### Phase 2

**Fig. 3:**
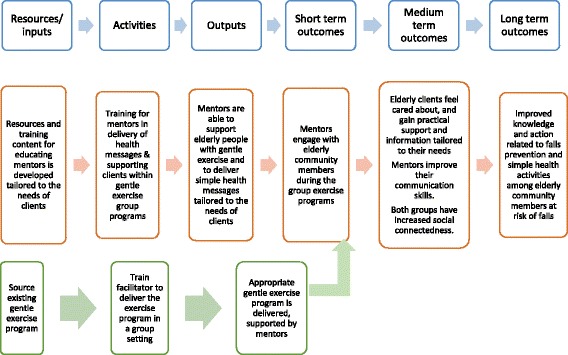
Example of a program logic model for training community members of a national women’s organisation as health mentors for elderly clients to reduce falls and decrease social isolation

From the pool of ideas generated during the co-design workshops, project team members at each site selected ideas that they considered could be used to achieve their project aims. Eight sites combined ideas to develop a single activity comprising a number of components while one site applied two entirely different interventions in different program areas. Program logic models were co-designed with each site. Figure 3 shows an example of a program logic model from Site #1.

The interventions that were determined from the program logic models varied in their design, approach and hypothesised mechanism of effect. An overview of interventions is shown in Table 3. Multiple intervention ideas arose in the workshops, and selection of the final ideas was driven by factors such as available resources, proximity of the intervention to the proposed outcomes, and endorsement of the intervention among staff at each site.

Interventions, shown in detail in Table 4, aimed to either improve clients’ health literacy directly or reorientate the organisation’s services and processes to make health information or services more accessible to people with diverse health literacy strengths and limitations. Overall, four distinct pathways were identified across the sites:

- three interventions targeted clinicians through provision of skills training and resources to support them to respond to a range of health literacy strengths and limitations in working to build clients’ capacity to self-manage their long term condition (sites 3, 8 and 9);
- two interventions utilised community volunteers to act as local health mentors and so build community members’ capacity to achieve better health outcomes (sites 1 and 4);
- three interventions aimed to directly improve clients’ health literacy through providing clients with resources or targeted training (sites 2, 5 and 7); and
- two interventions focused on redesigning existing service procedures to improve access to services for people with different health literacy strengths and limitations (sites 6 and 7).

In six of the nine sites, the aims of the final selected intervention aligned closely with the initial project aims. Differences were seen in the remaining three sites where initial project aims were less specific, with the final aim for these sites focused on specific client or organisational needs identified during the needs assessment and co-design workshops.

Rapid literature reviews for each intervention identified existing resources that were then used by two sites as the basis for developing client education materials to support their interventions, and in another case, highlighted useful strategies for engaging and training volunteers as heath mentors. Each site’s implementation and evaluation plan provided detail about the steps, processes and materials needed to apply their intervention, and the indicators required to evaluate its effectiveness. Approaches to measurement of short to medium outcomes, and where possible, long-term outcomes are detailed in Table 4. Evaluation of longer term outcomes was not possible given the relatively short time frame for implementation, but the program logic models identified shorter and medium-term outcomes as important intermediate achievements in producing longer term outcomes.

**Table 4:** Expected outcomes, evaluation activities and results for each of the 9 sites participating in Ophelia Victoria

Expected outcomes from program logic model	Evaluation activities	Participants	Results
Interventions utilising community volunteers			
Site #1 Metropolitan municipal council			
*Longer term*: Improved knowledge of falls prevention. Increased motivation to undertake health promoting behaviours; *Medium term*: Community members feel cared for; gain practical support and information; mentors improve communication skills and understanding of specific health problems; mentors and community members have increased social connectedness; *Short term*: Mentors engage with community members.	1) Evaluation of HLQ scales 2, 3 & 4 pre-post intervention in mentors and senior citizens (including Arabic speaking women’s group) 2) Satisfaction surveys – mentors; 3) Interviews with mentors and all clients.	8 mentors, 18 senior citizens participated in evaluation. Mean (SD) age of mentors = 69.8 (5.8) years, 100% female and 100% spoke English as their first language.	In HLQ scales, mentors showed small to large improvements with ES ranging from 0.26 (95% CI −0.73, 1.24) for scale 3 to 0.92 (−0.13, 1.94) for scale 2. For the senior citizens group, HLQ scores showed no improvement in scale 4 (ES 0.10 (−0.95, 1.14)). Interviews and focus groups with 18 senior citizens and mentors found most participants reported regularly applying what they learnt, increased mobility, and benefits from the social engagement. Mentors also reported an increase in their own confidence to support others and all reported a desire to continue in the mentorship role.
Site #4 Rural community health service			
*Longer term*: Increased community members’ capacity to navigate and engage with health services; improved health literacy and engagement of volunteers; increased social connectedness; *Short to medium term*: Community members are educated about the local health service, including navigation and engaging with GPs; reduced social isolation	1) Administration of HLQ scales 2, 5 & 6 at pre and post intervention with community members and volunteers. 2) Interviews with community members and volunteers. 3) Capturing of potential wider community effects via interviews.	14 mentors participated in training and evaluation; 7 community members participated in evaluation, with an estimated *n* = 100 reached by the intervention. Demographic data on participants not collected.	In the HLQ scales, participants completing both pre and post questionnaires (*n* = 18) showed moderate increases, with moderate ES ranging from 0.52 (95% CI −0.13, 1.16) for scale 5 to 0.56 (−0.09, 1.20) for scales 2 and 6. In interviews participants reported some GP's provided positive feedback on the *Good Questions form*. The form helped participants feel prepared and assertive during GP visits. *The Better Health Channel:* Improved awareness was evident. Some participants sought the help of a family member to gain access. *Using volunteers* who were active community members to deliver simple, word-of-mouth messages was reported as successful. Volunteers reported feeling useful and proud. Discussing one's health within immediate circles (family and community groups) reported frequently suggesting a ripple effect in terms of spread of the intervention's messages within existing circles
Interventions aimed at directly improving the health literacy of clients			
Site #2 Metropolitan municipal council			
*Longer term*: Increased management of health and adherence to recommendations; able to find out about supports/services and information as required; *Medium term*: Open and insightful exchange between clients and their GP; *Short term:* Clients use new skills and strategies during GP visits	1) Pre and post questions from HLQ scales 6 & 9. Scale scores not calculated as questions were modified. 2) A brief survey of the utility of the tool for clients 3) Focus group with assessment officers	8 clients completed modified HLQ scales pre-post intervention; 5 completed the utility survey. 88% were female; age >65 years. Focus group with four assessment officers	Overall client results showed slight increase in modified HLQ question scores. All 5 clients completing the utility survey felt discussions with the assessment officer about how to talk with the GP were useful. There were mixed responses to resources; some clients reported they were useful and others reported they were too long. Assessment officers reported that clients initially said they were happy with their relationship with their GP, but further questioning revealed many felt unheard by the GP. Case studies of positive outcomes when clients were encouraged to raise issues such as incontinence with their GP were discussed. Assessment officers reported being more aware of the need to question clients on this topic.
Site #5 Rural community health service			
*Longer term*: Clients are able to apply learnings to future situations; *Medium term*: Improved ability to find health information on the web; improved capacity to understand and appraise health information; *Short term*: Targeted participants (older adults) attend and or/or access information (wider community)	1) Administration of HLQ scales 1, 2, 5 & 7 pre and post-intervention (scale 1 as comparison in which no change expected); 2) Client interviews at 2–4 weeks post intervention 2 3) Number of people attending computer course	11 clients participated in intervention 1 (computer course), 27 in intervention 2 (presentation of DVD and checklist during planned activity groups). Pre-post HLQ scales collected on 32. Interviews with 12 participants from intervention 2. Demographic data not collected.	Changes in HLQ scales showed moderate increases with ES ranging from 0.43 (95% CI −0.07, 0.92) for scale 2 to 0.50 (0.00, 0.99) for scale 7. No change was seen in the comparison scale. Interviews with participants from intervention 2 found 4 participants reported an increase in using the internet to search for health-related information post-intervention. Barriers were not having a computer/internet and a lack of need for any health related information; 6 participants reported Increased levels of confidence or increased awareness in ability to appraise online information. The checklist was described as a useful resource
Site #7 Outer metropolitan community health service – Intervention 1			
*Longer term*: Community are optimally engaged with the service. Clients feel empowered to self-manage their health; *Medium term*: Increased awareness about the service; Staff are using a range of tools and strategies to engage and communicate with clients; The ‘My Health Diary’ is being used by 50% of eligible clients; *Short term*: Community engagement activities and promoting the service more broadly; Staff training around understanding the importance of health literacy and effective communication	*My Health Diary:* 1) Number of diaries taken; 2) Number of diaries being used, assessed by brief interviews with clients who consented to interview; 3) Clinician interviews	*My Health Diary:* 44 clients participated, 26 (62%) contacted for interview; mean age = 59 (17.0) years, 71% female; 92% with chronic condition. Interviews with *n* = 5 clinicians	*My Health Diary:* Of 26 clients interviewed, 6 reported using the diary. Interviews with clinicians found that staff felt uptake was low as diary was not formally promoted to clients, most of whom did not bring the diary with them to appointments. Different parts of the diary were felt to be more or less useful, with some replicating existing record systems. Two of the 5 clinicians interviewed reported the diary was easily understood by clients, who appeared to value having a concise record of health information.
Interventions focusing on developing health literacy skills of health personnel			
Site #3 Metropolitan community nursing service			
*Longer term*: Clients feel understood and supported by healthcare providers; clients have sufficient information to manage their health; clients understand health information well enough to know what to do; *Medium term*: Nurses integrate resources and techniques into everyday practice; *Short term*: Increased awareness of the resources and techniques among nurses; nurses have sufficient knowledge and confidence to apply appropriately	1) Administration of HLQ scales 2, 5, 9 pre and post intervention (scale 2 was comparison scale in which no change was expected) 2) The Diabetes Knowledge Questionnaire (DKN) (pre and post intervention) 3) Interviews with clinicians	24 clients participated in the intervention; 15 provided pre-post HLQ data. Mean age 75 (13.2) years, 67% female. Mean years with diabetes 9.8 (9.5), 96% had type 2 diabetes. Interviews with 9 clinicians	Client results for pre and post HLQ scales showed no improvement with ES of 0.08 (95% CI −0.64, 0.79) for scale 9 to 0.15 (−0.57, 0.87) for scale 5. Change in scale 2 = 0.04 (−0.67, 0.76). DKN scores indicated a small trend of improvement (ES = 0.24 (95% CI −0.43, 0.79). Interviews with clinicians found the diabetes education checklist was user-friendly and helped staff reframe education content/delivery to suit needs of individual clients. Using teach-back helped staff identify clients’ learning requirements and built a rapport. Using the learning styles tool reinforced the importance of the learning trajectory to both clinicians and clients. Staff discussed case studies of clients who became more proactive, asked more questions or showed improvements in self-management of their care.
Site #8 Metropolitan Hospital Admission Risk Program			
*Longer term*: Optimal use of health services by clients, preventing readmissions; *Medium term*: Clients have increased confidence to self-manage health and health crises; *Short term*: Improved client capacity to understand and use new health information and navigate health service.	1) Pre and post questions from HLQ. Scale scores not calculated as questions were modified; 2) Identification of client learning preferences; 3) Interviews with participating clinicians	In total, 70 clients participated; mean age = 76, 49% female, mean number of health conditions = 3. Interviews with clinicians (*n* = 8)	Preferred methods of learning information were: Talking through with someone (83%); writing down (53%). Least popular methods were brochures (33%) and pictures or diagrams (26%). Preferred methods for receiving information were face to face (93%). Email was least preferred (9%). Interviews with clinicians found teach-back 1) ensures client has an accurate understanding of what they need to do; 2) identifies gaps in clients' understanding; and 3) allows for better rapport between client and clinician. The health service navigation plan provided clients with a better knowledge of their services at the point of discharge. The learning styles tool was useful particularly for identifying clients with reading and language issues.
Site #9 Regional metropolitan Hospital Admission Risk Program			
*Longer term*: Increased appropriate demand for early intervention health services; *Medium term*: Improved client capacity to understand and appraise new health information relevant to their needs; Increased confidence to self-manage health and health crises; increased capacity to effectively and appropriately engage with health services and providers; *Short term*: HARP clinicians collaborate with clients	1) Administration of HLQ scales 2, 4, 8 pre and post-intervention (scale 4 as comparison in which no change was expected). 2) Interviews with clients 2) Focus group and interviews with clinicians	48 clients completed the HLQ pre-post intervention; 11 participated in the interviews; mean age 63.9 (15.7) years; 45% female; mean number of health conditions 6.3 (4.3); 11 clients and 10 clinicians participated in interviews	Changes in HLQ scales showed no to small increases with ES ranging from 0.02 (95% CI −0.41, 0.45) for scale 2 to 0.24 (−0.19, 0.67) for scale 8. No change was seen in the comparison scale (scale 4). Findings from the client interviews showed clients felt comfortable with the experience and with showing their understanding through actions or words. 4 clients expressed confidence using the appointment planner and reported it was a helpful resource. Clinician interviews found the benefits of using teach-back were: 1) allows clients to take more ownership of their health; 2) builds on client's capabilities; 3) revealed clinicians’ misconceptions about client's level of understanding. The appointment planner was used less often. Clinicians noted it was a useful tool, but needed to be embedded into their practice. Clients appeared to have their own systems of managing appointments, although forgetfulness played a prominent role in recalling appointments. The Learning Styles Tool was praised by clinicians who felt it alerted them to client's literacy needs, and allowed tailoring their practice to the client's requirements. Others felt it helped focus on client preferences in contrast to clinician's expectations and assumptions.
interventions focused on redesigning existing service procedures			
Site #6 Metropolitan community health service			
*Longer term*: Increased access and links with local health services; strengthened relationship, trust and engagement with local health service; *Medium term*: improved client access local health services; *Short term*: Referral pathways between services are developed and clinicians undertake referrals.	1) Focus group with central intake staff; 2) Telephone survey with dental clients	7 clients, 3 dentists and 3 intake staff participated in the study and evaluation activities. Telephone survey with 7 dental clients. Demographic data not collected on clients. Focus group with 3 central intake staff	Telephone survey with dental clients indicated all clients were comfortable with the dentists raising health issues, and all thought the intervention was a good idea. In total, 4 clients were referred to new services of which 3 were pleased with the outcome. One person reported waiting a long time for their initial appointment with the primary health service provider. Focus group with central intake and dental staff found the referral process between dental services and primary health care was efficient and not overly time-consuming. Staff reported the process increased clients' awareness of services available to them.
Site #7 Outer metropolitan community health service – Intervention 2			
*Longer term*: Improved quality of life and health outcomes; *Medium term*: Increased capacity of clients to navigate the healthcare system resulting in early response to declining health to prevent unplanned readmission. *Short term*: Increased knowledge of clients in engaging with the health system	*For Care coordination:* 1) Client case studies and interviews; 2) Clinician focus group	*Care coordination:* Focus group with 4 staff,	*Care coordination:* Staff focus group found the intervention avoided the need to repeatedly question clients and allowed recording of case-management information more efficiently, especially for short term clients with more acute needs. A case study of one client found that over 8-months, 22 episodes of care coordination were documented by 5 separate nurses, resulting in closer engagement with the GP and avoidance of one hospital admission

Abbreviations: *ES* Effect size, *SD* standard deviation. Scales of HLQ are: 1) Feeling understood and supported by healthcare providers; 2) Having sufficient information to manage my health; 3) Actively managing my health; 4) Social support for health; 5) Appraisal of health information; 6) Ability to actively engage with healthcare providers; 7) Navigating the healthcare system; 8) Ability to find good health information; and 9) Understand health information enough to know what to do

Sites each undertook between one and three PDSA quality cycles to refine processes and materials. In most cases the refinements made were small. For example, one site developed prompts to remind clinicians to use teach-back, while another identified the need to develop locally-based training videos and filmed these themselves using hand held devices. A third site decided to limit the scope of their intervention by initially delivering it within group-based programs, with plans to extend it to home-based clients at a later stage.

### Phase 3

Evaluation findings for each of the interventions tested are shown in Table 5. Across the nine sites, 228 clients (range 5 – 70) and 22 volunteers (range 8 – 14) participated in evaluation activities including completing pre-post HLQ scales, interviews or focus groups. Forty-two staff (range 4 – 10) involved in delivery of the interventions were also interviewed.

As shown in Table 5, all evaluations used a quasi-experimental (pre-post) design, with five of the nine sites utilising a mixed-methods approach. Qualitative data, obtained through interviews and focus groups with 92 clients and volunteers (range 5 – 26 across sites) and 43 clinicians (range 3 – 10) showed small, but positive impacts for clients, volunteers and clinicians, indicating that for the most part, shorter-term outcomes from the site program logic models were achievable. In the seven sites that also undertook quantitative analysis, findings supported qualitative data to varying degrees. Effect sizes (ES) for individual HLQ scales was nil/minimal (ES 0.02, 95% CI −0.41, 0.45) in two sites, moderate in two sites, and moderate/large in one site (ES 0.92, 96% CI −0.13, 1.94). Where comparison HLQ scales were used (i.e. a scale where no change was expected), small or no changes were observed.

For the three sites with interventions directly targeting health personnel, the interviewed staff reported increased awareness and responsiveness to clients’ health literacy-related needs and improved clinician-client rapport, particularly where teach-back [20] was used. For these interventions, two sites administered selected HLQ scales to participating clients, with no/small improvements seen. For the two sites with interventions that trained volunteers to act as health mentors, the volunteers themselves reported increased confidence to support others and a sense of feeling useful. In both cases, clients or community members receiving these interventions reported positive changes in behaviour. Small, moderate and large improvements were seen for selected HLQ scales in both sites. Three sites developed interventions directly targeting clients’ health literacy through providing resources or education. Findings from these sites were mixed. Some clients and clinicians reported that resources were not relevant to their needs, while others indicated they were useful. Only one site (site #5) evaluated changes in selected HLQ scales, observing moderate improvements. For the two interventions in which existing service procedures were redesigned to improve access to services, evaluation data were limited. However, qualitative findings for these two sites indicated that the process of redesign was feasible and appropriate.

Evidence for operationalisation of the eight Ophelia principles was explored across the nine sites (see Table 5) and evidence was present for each. Being *outcomes focused* (principle 1 [P1]) was part of the initial process of engagement and a primary aim for sites. This was reinforced through the use of program logic models. Each site was *equity driven* in that all sites formally considered which clients may not be receiving the full range of services or not achieving optimal health outcomes [P2]. The workshops to generate intervention ideas drew on the *local wisdom* of local stakeholders [P3, P5] ensuring *co-design* took place. The use of the HLQ to inform the vignettes ensured sites focused on *local health literacy needs* [P4, P7]. Organisations generated intervention ideas that ranged from those directed at individuals through to the engagement of external agencies, i.e., ideas were generated and applied, where relevant, across *all levels of organisations* [P8]. For some organisations the Ophelia improvement processes have been continued to ensure their *interventions remain relevant and effective* [P6] however longer term follow-up is required to confirm *sustainability* of the interventions developed during the project.

**Table 5:** Evidence for application of the Ophelia principles

Ophelia principle	Evidence of application	Limits, difficulties, lessons learned
1. Outcomes focused	Phase 1: establishing project aims that were focused on improving health outcomes either in vulnerable clients or that took a population-based approach to selection of the target group; Phase 2: use of program logic models, which by their nature are outcomes focused, Phase 3: ensuring these logic models were used as the basis of implementation and evaluation plans and that evaluation measures captured these outcomes where possible.	Most sites had some difficulty defining a specific target group at the start and narrowed their focus during the project. Tools were developed during the project to assist selection of a focus.
2. Equity driven	Design process deliberately focused on clients who may not be receiving the full range of services or not achieving the full range of outcomes. Examples include: • embedding brief interactive health literacy screening into a service’s assessment process to identify *all* clients experiencing difficulty engaging with health providers, • developing a process that enables low-income clients to access a primary health service. While not directed to do so, many sites incorporated elements that made them available to disadvantaged groups as well as to core client groups. The process steered clinician’s thoughts towards equity including through use of vignettes that focused on how the health literacy profile could cause people to miss out.	There can be difficulties ensuring an adequate number of responses to the HLQ from people most at risk, especially people who have very little engagement with health services. Collaborative and outreach projects to collect data from high risk groups in community settings may be a useful supplement.
3. Co-design approach	Co-design was inherent in all activities; from data collection and interpretation, to development and pilot-testing of intervention plans, through to conducting evaluation activities. In Phase 1, the process of engaging clinicians in ‘their’ data and interventions may have been enhanced by the use of narratives. This approach to presenting data was very engaging for clinicians and managers, generating multiple intervention ideas.	The researchers probably underestimated the time, training and support required for sites to work through all stages of the project. Many tools and exemplars have been developed to assist future users.
4. Needs-diagnostic approach	Health literacy and demographic data were collected from a pre-defined target group in whom health literacy was thought to contribute to inequitable service access or poor health outcomes. Collection of health literacy data using a comprehensive and robust measurement tool, designed for this purpose, supported this process. The multidimensional tool allowed identification of different profiles of strength and weakness rather than just single health literacy scores.	Potential contextual or other barriers to access should be considered in detail at the outset so that additional questions (other than health literacy) can be included in needs assessment.
5. Driven by local wisdom	The co-design workshops allowed local clinicians and managers to draw on their knowledge and provide intervention ideas in response to needs identified from the local data collection. Interventions were: tailored to local context (such as the outreach nature of interventions from rural organisations); took existing organisational processes into account (e.g. formalising the case-management role of community nurses), and; utilised existing resources (e.g. using volunteers already in place). During implementation of the interventions, use of quality improvement cycles allowed clinicians to refine and further tailor the intervention according to their local knowledge. Design of intervention evaluation was also driven by local knowledge, with project teams determining the most appropriate indicators for their client or community group.	Allowing time in the co-design workshops for detailed discussion of the issues raised within the vignettes is essential if solutions are to be responsive. At all stages of the process, involving the people who know the client group and who will be delivering the intervention is also essential.
Ophelia principle	Evidence of application	Limits, difficulties, lessons learned
6. Sustainable	Since completion of the study, several sites have commenced using existing organisational quality improvement processes to ensure their interventions remain relevant and effective. For some sites, interventions are seen as stepping-stones to broader objectives with plans to use small interventions at one level to build up over time to achieve organisational priorities and objectives. For example, the intervention to develop a client access point between dental and primary health services will be used as the basis for developing an organisational policy on service access within 5 years.	Having management visibly support the project from the start helped ensure continuity of the intervention at some sites. External factors (such as changes to the chronic disease funding model) influenced sustainability.
7. Responsiveness	Responsiveness was considered in terms of how the organizations responded to health literacy diversity and other unique needs in the target population. It was most clearly demonstrated through use of cluster analysis to capture the diverse range of health literacy profiles. In relation to diversity in the delivery of interventions, three sites selected similar activities (teach-back and learning styles assessment). However, these interventions were applied to achieve different outcomes. Any large or diverse organisation seeking to apply the Ophelia process will need to consider that health literacy will vary considerably between clients, and apply the process accordingly. For example, organisations with different cultural groups using their services may need to collect sufficient needs assessment data to ensure that diversity is adequately captured, and then will need to tailor interventions to these different groups’ needs, or in some cases, develop specific interventions.	More recent Ophelia projects have conducted co-design workshops with consumers or community members, separate to those held with clinicians, but using the same vignettes. This ensures consumers’ perspectives are included. More than one workshop may be needed if there is important cultural or other diversity in the target group.
8. Systematically applied	We have previously identified that health literacy is a potential barrier at multiple access points within a service [7]. Health literacy interventions are therefore required at all levels of client engagement [26]. In this study, interventions focused on multiple levels including: • directly targeted at improving individual client’s skills, • enabling clinicians to respond appropriately to health literacy needs (existing clients, clients who approach the service, community outreach), • changes in organisational processes, • engagement with external agencies Several interventions encompassed more than one level.	Using these 4 levels to categorise the intervention ideas helps to demonstrate how an intervention can be refined to encompass more than one level. Some recent Ophelia projects have also incorporated a further workshop to discuss and select interventions; these workshops can include representatives from external agencies and funding bodies.

## Discussion

We have described a systematic process designed to enable health services to identify and respond to the health literacy strengths and needs of their clients. Nine different sites were able to collect health literacy data, take part in co-design workshops, use program logic models, apply quality improvement cycles, and then implement and evaluate innovative interventions. The evaluation data indicate that the Ophelia process is a feasible approach by which organisations can develop tailored responses to the health literacy needs of their clients. While quantitative impacts were generally small, consistent positive findings from qualitative data indicated that service redesign occurred and some short-term outcomes for selected interventions were achievable. Impacts upon HLQ scale scores were less consistent, which may be related to the short time frame available for implementation of interventions.

**Fig. 4:**
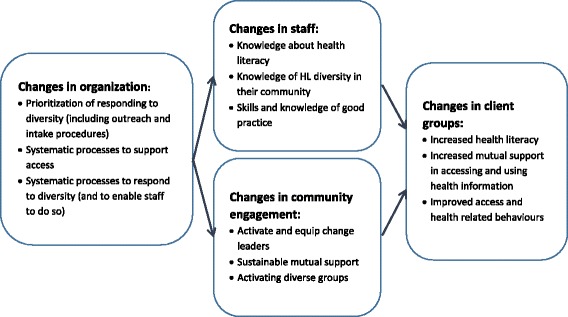
An integrated framework for health literacy interventions

In examining the program logic models and hierarchies of short, medium and long-term outcomes identified by the sites, it is possible to build an integrative program logic of changes occurring at different levels of the health system that allow it to be more responsive to the diversity of health literacy strengths and weaknesses in the community (Fig. 4). This framework recognises that while the main focus of planning and intervention may be at one level, success or failure is dependent also on what occurs at other levels: organizational change must be manifest in practice change in personnel or engagement with new partners in the community; changes in the activities of staff must be authorised and enabled by accommodations in the organization and must produce changes in the experiences of clients and so on. While it is possible for the primary focus to be on any of the levels indicated, planning, activities and monitoring at other levels are required to enable these changes to occur.

In considering the impact of the different interventions, it is also necessary to consider the degree of newness that the intervention introduced to existing practice in the organization. In general, the interventions that utilised community volunteers and those that attempted to directly improve the health literacy of clients involved a greater change from normal practice, than did the interventions focused on skills of health personnel or on changing organizational processes. For example, the interventions to develop skills of health personnel occurred in programs where staff already had a clear role and a high level of skill in health education. In these contexts, the intervention may best be considered as a quality improvement activity, and/or an activity for skill development of new staff. However, it may be just as appropriate to improve relevant knowledge and skills in those organizations where staff have lower levels of commitment to high quality client education.

While the size size and composition of the samples included in the evaluation do not allow us to generalize broadly, those interventions which focused on community engagement and directly changing the health literacy of clients were more likely to lead to moderate to large changes in the targeted HLQ scales. This cannot be taken to mean, however, that those interventions are always to be preferred. As noted they tended to be more novel in terms of engaging new target groups in new ways to improve access and equity. Generally, they were also more labour intensive and probably more expensive interventions. Furthermore, interventions that focus on staff skills and organizational change are likely to have a broader impact for all users of an organization’s services.

This is one of very few studies that have shown improvements in scores on any standardized health literacy measure. The largest systematic review of health literacy studies ever conducted by the US Institute of Medicine [21] did not look at changes in health literacy scores as an outcome but rather focused on other outcomes for people with limited health literacy (e.g., knowledge, behaviour change). A review by Taggart et al. identified many changes that were classified as broadly health literacy changes but which were mostly changes in knowledge or management of specific diseases, changes in self-efficacy for target behaviours, or changes in behavioural intent related to stages of change in target behaviours [22]. Some studies focusing on health literacy for mental health [23] and oral health [24] have demonstrated changes in knowledge or attitudes, and a study of consultation skills training demonstrated improvements in interactive health literacy [25]. The improvements shown in this present study may be a result of greater sensitivity of the HLQ compared with other scales used in clinical settings, due to the broader range of concepts that it measures and the provision of scores for each independent scale.

This study has also demonstrated that sites were able to select scales likely to change based on the program logic of their interventions. Participating organizations demonstrated some success in selecting a limited number of scales in order to assess health-literacy-related intermediate outcomes while using a comparison scale that was not expected to change.

In this study, proof-of-concept was defined as successful application of the eight underlying principles to achieve the development of health literacy-informed interventions to impact on health and equity outcomes. Evidence indicating that the Ophelia approach was applied with fidelity is shown in Table 5. Overall, the Ophelia principles were operationalised at many levels of the project across all sites.

This proof-of-concept study of the Ophelia approach aimed to generate data to develop, improve and apply health literacy interventions in real world settings. Although further analysis is required to identify the determinants of successful implementation, at the organisational level, one determinant appeared to be the early establishment of a clearly defined project objective. Another determinant may be having ‘flexible’ interventions that can respond to different needs of clients, to changes in the organisation, or to feedback from clinicians. This flexibility is essential if quality improvement cycles are to be effective, and may be an important characteristic of ‘responsive’ health literacy interventions. Finally, having a suite of activities rather than one fixed intervention seemed to be an important determinant, possibly because it allowed clinicians to use their own discretion in tailoring what they do for individuals or groups of service users.

An important strength of this study is the application of a co-design process that emphasised participatory design of interventions through genuine engagement of practitioners and managers from across prevention and care pathways. Great care was taken to elicit and utilize their expertise and local knowledge, with the vignettes providing an effective vehicle for this in the co-design workshops. As intended, the workshops generated locally relevant, and for the most part, implementable interventions. The co-design approach specifically sought to assign increasing ownership and responsibility of the application of the intervention to sites. The intensive engagement of local personnel in all stages was, however, time consuming for some stakeholders due to the number of steps requiring feedback and local decision-making. There was also a risk of losing the overall focus in attending to details at each step. The Ophelia process sought to maintain a balance between these elements, and program fidelity was reasonably achieved according to the utilisation of Ophelia principles.

An important marker of success was the retention of all nine sites throughout the entire study period. Almost all sites and project teams experienced project team-related or organisational change, with only four of the sites retaining the original project team members over the three years of the project. Two sites amalgamated with other services, and two sites underwent major organisational re-structuring. Despite this, all sites continued active participation in the study, and in most cases expressed a sense of ownership of ‘their’ intervention and a desire to generate their intended objectives and complete the project. The observation that interventions were readily taken up within organisations even in the context of time pressures and financial constraints experienced by most health services suggests they were acceptable to front line providers and their managers (data not shown).

Limitations of the study include the relatively short time-frame of the project, which meant that the longer-term impacts of interventions were not able to be assessed. Nevertheless, short-term outcomes described in the logic models for all sites were met, suggesting accuracy in the thinking that occurred about the mechanisms by which each intervention worked. Small sample sizes for phase 3 mean that in most cases, quantitative data analyses are only indicative. As this was a feasibility study, and we were not seeking to formally test hypotheses, we used effect sizes with 95% confidence intervals. Generally, the confidence intervals were wide (reflecting the small sample sizes and the variable responses of individuals tested). Nonetheless, some interventions generated moderate mean effects at the individual level alongside clear indications of clinician and organisation change. It is important to note that the client-level pre-post changes are potentially influenced by a range of biases, and there were no control groups. While the application of a ‘comparison’ HLQ scale (where no change was expected) generally provided some evidence the interventions generated intended effects, future formal evaluations will be greatly strengthened through more comprehensive evaluation designs. A further potential limitation is the generalisability of the Ophelia approach in other settings and countries. Of note, the approach is currently being applied in a diverse range of healthcare services and communities in the UK, Thailand and Norway, where community members contribute significantly to the co-design approach.

## Conclusion

The Ophelia process was successfully applied across nine sites with evidence of successful generation of a wide range of health literacy interventions directly related to organisations’ priorities. Proof-of-concept was evidenced through successful application of eight a priori principles. The co-design elements, with a wide range of stakeholders across disparate organisations, generated locally relevant and fit-for-purpose interventions that were implementable and achieved outcomes at four key levels, including organisational level process improvements, improvements in staff knowledge and skills, improvements in community engagement, and direct improvements in client outcomes and equity.

## Additional files

Additional file 1: Phase one interviews.pdf (template for client interviews used to inform development of vignettes). (PDF 348 kb)
Additional file 2: Implementation plan template.pdf (template for implementation and evaluation plans used in Phase two of the Ophelia process). (PDF 346 kb)
Additional file 3: Phase three interviews.pdf (template for client, volunteer and practitioner interviews conducted in phase three). (PDF 382 kb)

## Abbreviations

ES: Effect size
HACC: Home and Community Care
HLQ: Health Literacy Questionnaire
Ophelia: Optimising health literacy and access
PDSA: Plan, Do, Study, Act
