# Supplementary material for: Systematic development and implementation of interventions to OPtimise Health Literacy and Access (Ophelia)
Source: BMC Public Health. 2017 Mar 3;17:230. doi: 10.1186/s12889-017-4147-5 (PMC5335493; doi:10.1186/s12889-017-4147-5)
Supplement: Additional file 1: — Phase one interviews.pdf (template for client interviews used to inform development of vignettes). (PDF 348 kb) [file 12889_2017_4147_MOESM1_ESM.pdf]

Systematic development and implementation of interventions to OPtimise Health Literacy and Access (Ophelia)

Additional file 1: Phase one interviews (template for client interviews used to inform development of vignettes)

Purpose of the interviews:

To get a sense of the client's 'journey' with their health condition, especially in recent times, and how things have changed for them over the past two years.

Interview preparation:

Establish background to patient health journey, e.g., time of diagnosis, information and chronic disease management then and now.

Determine patient's dominant health literacy needs, as identified by the HLQ (i.e., domains with relatively lower HLQ scores).

Determine the health literacy areas the patient feels confident about, as identified by the HLQ (i.e., domains with relatively higher HLQ scores).

Background questions

1. Can you please tell me when you were diagnosed with your health condition?

2. Can you tell me a bit about what it was like for you to manage your condition in the time after you were first diagnosed?

3. Do you feel you had enough information about your condition back then? Where did you get your information?

4. Can you please tell me what it is like for you to manage your condition now?

5. Do you feel you have enough information about your condition now? Where do you get your information?

6. Thinking about your health, how have things changed for you over the past two years

**Patient-specific strengths and challenges as indicated by the HLQ** [Choose the highest or lowest scales – don't necessarily need to talk about all of these.]
**Scale 1. Feeling understood and supported by healthcare providers**<br>*- Your HLQ scores indicate you have good relationships with your Healthcare providers. Has it always been this way? What has helped you to have a strong relationship with your Healthcare providers?*<br>***Or***<br>*- Your HLQ scores indicate that you've found it difficult to establish good relationships with Healthcare providers. Has it always been this way? What would help you to improve these relationships?*
**Scale 2. Having sufficient information to manage my health**<br>*- What helps you to feel you have enough information to manage your health and make decisions?*<br>***Or***<br>*- What would help you to feel confident that you have enough information about your health?*

**Scale 3. Actively managing my health**<br>*- How did you learn to manage your health? What do you do to manage your health?*<br>***Or***<br>*- What do you need to help you manage your health differently?*
**Scale 4. Social support for health**<br>*- What has helped you to build a strong system of support from family and friends?*<br>***Or***<br>*- What would help you to build relationships with family or friends?*
**Scale 5. Appraisal of health information**<br>*- What helps you to understand health information? How do you work out what is best for you?*<br>***Or***<br>*- What would help you to understand health information and work out what is best for you?*
**Scale 6. Ability to actively engage with healthcare providers**<br>*How comfortable do you feel about talking about your health with Healthcare providers? What has helped you with this?*<br>***Or***<br>*- How comfortable do you feel about talking with Healthcare providers? What would help you to feel more comfortable to talk with them about your health?*
**Scale 7. Navigating the healthcare system**<br>*- How do you find out about the health and support services that you need?*<br>***Or***<br>*- What would help you to find out about health support services that you need?*
**Scale 8. Ability to find good health information**<br>*- What has helped you to find information about your health?*<br>***Or***<br>*- What would help you to find information about your health?*
**Scale 9. Understanding health information well enough to know what to do**<br>*- What has helped you to understand written health information?*<br>***Or***<br>*- What would help you to understand written information about your health?*
