# Supplementary material for: Systematic development and implementation of interventions to OPtimise Health Literacy and Access (Ophelia)
Source: BMC Public Health. 2017 Mar 3;17:230. doi: 10.1186/s12889-017-4147-5 (PMC5335493; doi:10.1186/s12889-017-4147-5)
Supplement: Additional file 2: — Implementation plan template.pdf (template for implementation and evaluation plans used in Phase two of the Ophelia process). (PDF 346 kb) [file 12889_2017_4147_MOESM2_ESM.pdf]

Systematic development and implementation of interventions to OPtimise Health Literacy and Access (Ophelia)

Additional file 2: Implementation and evaluation plan template

Intervention aim:

To prepare the intervention we need to:

Development Activities

(e.g. deliver training session to 3 staff, evaluate and modify, then repeat training to 3 new staff)

Who will undertake these preparation activities:

Responsibilities

To make the intervention work we need:

Intervention Inputs

(e.g. training materials and content of training session)

The intervention is:

Intervention Process (intervention description and timeline

Intervention Description

(e.g. Deliver 60-minute training sessions to all staff in use of teach-back and simplified education strategies)

What we want to achieve:

Outputs *(intervention access and services readiness. Intervention quality and safety)*

……program delivered such that:

(e.g. ….mode of delivery is matched to client's health literacy needs & learning preference)

Outcomes - short and medium term *(coverage of interventions, prevalence of risk behaviours and factors)*

(e.g. Patients' ability to identify how they will self-manage their condition is improved

Impact/ longer-term outcomes *(improved health outcomes, improved equity, responsiveness, efficiency)*

(e.g. All people in target population are educated according to their needs)

When tasks will be completed:

Timeline

How we could assess feasibility of intervention:

Feasbility measures

e.g. staffing time & costs

How we could assess delivery of intervention:

Process outcome measures

e.g. response rate (patient and clinician)

How these achievements could be assessed:
