# Supplementary material for: Systematic development and implementation of interventions to OPtimise Health Literacy and Access (Ophelia)
Source: BMC Public Health. 2017 Mar 3;17:230. doi: 10.1186/s12889-017-4147-5 (PMC5335493; doi:10.1186/s12889-017-4147-5)
Supplement: Additional file 3: — Phase three interviews.pdf (template for client, volunteer and practitioner interviews conducted in phase three). (PDF 382 kb) [file 12889_2017_4147_MOESM3_ESM.pdf]

Systematic development and implementation of interventions to OPtimise Health Literacy and Access (Ophelia)

Additional file 3: Phase three evaluation – interview questions by Site (templates for client, volunteer and practitioner interviews conducted in phase three)

**Site #1 interview schedule for senior citizens and mentors, 4 weeks following completion of exercise program.**<br>**Purpose of the interview:** To investigate senior citizens and mentors' perceptions of the exercise program, and any changes to their confidence regarding their mobility, whether they are using the information provided in the exercise group, the spread of messages in their community, and any changes to their social engagement.
1. Can you tell me about sorts of things that would happen in the exercise group?<br>2. Thinking back to before you started the program, how confident were you with your mobility – that is, with things like walking, or getting around generally?<br>3. How confident are you with your mobility now?<br>4. Who have you spoken to about the program (how many people and who were they?)<br>5. What sort of things did you speak to these people about?<br>6. How are you using the things you learnt in the program now? If you are not using the things you learnt, why is this?<br>7. Are you involved with any organisations / group activities now that you were not involved with before the exercise program? If yes, when did you join these organisations and why?<br>8. Is there anything that you would change about the group or the exercise program?<br>9. Is there anything else you would like to say about the program?

**Site #3 interview questions for practitioners, 2-4 weeks after completion of diabetes education intervention.**<br>**Purpose of the interview:** To investigate perceptions about the value of the diabetes educational intervention, any changes to their practice, and any impact on their clients' self-management behaviour or understanding.
**Delivery and reach of the intervention/ early evidence of effect**<br>1. Can you describe the Ophelia intervention at your organisation?<br>2. What, if anything, has changed for you since participating in Ophelia? In what ways are you thinking or acting differently now? What is <u>the most</u> significant change?<br>3. What evidence has led you to believe that the Ophelia intervention has caused these changes in your thinking or clinical practice?<br>4. What sort of challenges did you come across when delivering education to your clients using the teach-back method of education and the diabetes education tool?<br>5. How useful was this intervention in improving your clients' ability to self-manage their diabetes? Why do you say this? What sort of changes have you noticed in your clients' behaviour as a result of this intervention? *Prompt: what else might explain this, is this different to clients not in the study?.*<br>**Implementation/ training**<br>6. What was involved in the training for this intervention?<br>7. How useful was the training? Why do you say this? *Prompt – how might it have been improved*<br>8. In addition to the training, what sort of support did you receive to be able to put the teach-back and diabetes education intervention into practice?<br>**Lessons learned and critical success factors**<br>9. What do you see as critical factors that will help you continue to use the diabetes education tool and teach-back?<br>10. Are there any resources or tools that have been particularly helpful in supporting you to use the education tool and teach-back?

Site # 4 interview questions for volunteers, 3-4 weeks after delivery of health messages in their community.

Purpose of the interview: To investigate volunteers' perspectives on their involvement in the community volunteer program including training, delivery, early outcomes and any challenges.

Training

1. How did you find out about the training? What made you decide to become involved?
2. What did you like most about the training? What did you like least?

Delivery and reach of the intervention/ early evidence of effect

3. Could you describe the specific health messages that you were asked to talk to your group about?
4. Were you able to deliver the the My Questions for Good Health and Better Health Channel messages to your group? If you did deliver the messages, can you tell me how it went? Were there any challenges in delivering the messages? Can you give me some examples of this?
5. If you weren't able to deliver the message, could you tell me what prevented this or what would have helped you do this?
6. Can you tell me who you talked to about the messages and where you did this? Have any of these people told you that they have spoken to others about the messages?
7. Has anyone else mentioned to you about these messages/ or the Ophelia project in general? Can you remember what they might have said to you?
8. Did anyone you talked with say that talking to health providers is difficult for them? Has anyone told you this has changed at all since hearing your health messages?
9. Did anyone you talked with say that finding reliable health information is hard? Has anyone said this has changed at all since hearing your health messages? Can you give some examples?
10. What sort of things have people told you about how they used the messages you gave them? Any feedback from people using the My Good Questions for Good Health form or Better Health Channel website?
11. What, if anything, has changed for you since being in this study?

Lessons learned

12. Would you do this again? Why or why not?

Site #4 Interview questions for clients, 3-4 weeks after having received health messages from volunteers

Purpose of the interview: To investigate clients' perspectives on their involvement in the Ophelia project including the value of the messages/resources, and any early outcomes.

Delivery and reach of the intervention/ early evidence of effect

1. Can you remember what the messages [*volunteer's name*] gave you were about?
2. How did you feel about hearing these messages in your [*tai chi, quilting etc.]* group? Was there any discussion in the group about the messages? Can you describe this discussion?
3. Have you shared the messages you had heard from the volunteer with anyone else? Can you tell me a bit about where you did this, and what your relationship to this person is?
4. Has anyone else mentioned to you about these messages/ Ophelia?
5. Thinking back to before you heard the messages, what was talking with health providers like for you? Can you give me some examples of this?
6. How do you feel now about talking with health professionals? What has changed? Why do you think this has or has not changed?
7. Have you used the My Good Questions for Good Health form at all? In what way and with whom?
8. Had you heard about the Better Health Channel before this? Have you used it since hearing about it? In what way have you used it?

Lessons learned

9. Can you think of examples in the future of when you might use the Good Questions for Good Health form or ask questions of your health professionals a result of being in this project?
10. Would you use Better Health Channel in the future? Can you think of an example of when you might use it?

**Site #5 interview questions for clients, 4-6 weeks after attending the computer training course or seeing the DVD about accessing reliable health information online**<br>**Purpose of the interview** to determine whether the computer training/ DVD helped them feel more comfortable about looking for health information online and how useful the intervention was to them.
**Context/ background**<br>1. Can you tell me what you remember about the video/ computer training session?<br>2. Thinking back to before you did the training / saw the DVD, what were some of the ways you would find information out about health? *('for example if you wanted to eat more healthily, where would you have found out information about how to do this?')*<br>**Delivery of the intervention/ early evidence of effect**<br>3. Since you did the training / saw the DVD have you looked for health information online? Can you give me some examples of this?<br>4. Would you say there has been any change in the number of times you have gone online to look for health information since doing the course?<br>5. How confident do you feel about using the internet to find good quality health information?<br>6. How about confidence with this method compared to finding good quality health information from other sources?<br>7. Have you used any information you have found online? Can you give me some examples of this?<br>**Lessons learned**<br>8. Thinking back to when you did the training/ saw the video, is there anything that could be done differently next time?

**Site # 7 interview questions for clinicians, 4 weeks after using intervention 1 – My Health Diary (MHD) - with clients**<br>**Purpose of the interview**: To obtain feedback from clinicians on usefulness of MHD for clients and clinicians, any identified barriers to client's uptake of tool, and any suggestions for ways in which use of the tool can be enhanced.
**Introducing the diary to the clients**<br>1. How easy was it for you to introduce the diary to clients?<br>2. How difficult was it to explain how to use the diary? How much time did it take? Do you have any suggestions for ways in which this stage could be improved?<br>3. How easy was it to document the use of the diary in the client history? Were there ways in which this could have been done better?<br>**Client uptake of the diary**<br>4. What were some of the ways that clients used the diary? Were there parts of the diary that clients used more than others?<br>5. What were some of the client-related barriers to using the diary?<br>6. What did clients like about the diary?<br>7. How well did the clients engage with the diary? Is there any anecdotal evidence to support this?<br>**Usefulness of diary from clinician's perspective**<br>8. How useful was the diary to you as a clinician?<br>9. How often on average did you look at or write in the diary?<br>10. Did using the diary change your practice with that client in any way?<br>**How could the diary be improved?**<br>11. In what way could use of the diary be improved?

**Site # 7 interview questions for clients, 4 weeks after taking My Health Diary from the service's waiting room**<br>**Purpose of the interview**: To determine how the clients felt about using the My Health Diary
1. How did you feel about using the My Health Diary with your community health clinician?<br>2. What did you like about the diary?<br>3. What didn't you like?<br>4. How would you change it?<br>5. How easy will it be for you to use the diary with other clinicians, such as your GP?
**Site # 7 interview questions for clinicians, 4 weeks after completion of intervention 2 - care-coordination project**<br>**Purpose of the interviews**: To obtain feedback from clinicians on the usefulness of the care-coordination process, any identified barriers to implementation of the process and suggestions for improvement.
**Starting off**<br>1. How easy was it for you to start using care coordination in practice?<br>2. Do you have any suggestions for ways in which this stage could be improved?<br>**Resources and guidelines**<br>3. How useful was the activities checklist to you in practice?<br>4. Were there ways in which the resources and guidelines could be improved?<br>5. How easy was it to document the use of care coordination in the client history?<br>6. Were there ways in which this could have been done better?<br>**Usefulness of care coordination process**<br>7. How well does care-coordination work for you and your clients?<br>8. How much time does it save you and other clinicians?<br>9. Are there better outcomes for clients? Is there any anecdotal evidence to support this?<br>10. In what ways has using the process impacted upon your clinical practice?<br>**How could the process of introducing and using care coordination be improved?**<br>11. In what way could this process be improved?<br>12. Would additional resources or supports be helpful?<br>13. Would there be potential to introduce care coordination across the organisation?

**Site #8 interview questions for clinicians, 4-5 weeks after using teach-back, learning styles tool and navigation plan with clients**<br>**Purpose of the interview:** To investigate clinicians' perspectives on their early involvement in the Ophelia project including delivery, early outcomes and any challenges
**Context/ background**<br>1. How would you describe the Ophelia intervention in your organisation? What were the specific elements?<br>**Delivery of the intervention/ early evidence of effect**<br>2. Can you give me some examples of how you have used teach-back in your practice? How frequently are you using it?<br>3. I'm particularly interested in finding out about strategies that are helpful with more complex clients, or challenges you have come across and ways you have got around these. Can you give me any examples of these?<br>4. What sort of things might have stopped you from using teach-back? Can you give me some examples of this?<br>5. Can you tell me about the learning styles tool. How have you been using that? Can you give me some examples?

6. If you have used it, how did you adapt your teaching approach to meet your client's preferred learning style? Any examples?
7. What sort of feedback have you had from clients who used the learning styles tool?
8. Can you give me some examples of how and when clients have used the navigation planner?
9. How many clients used the navigation planner?
10. What sort of feedback have you had from clients who used the navigation planner?

Lessons learned

11. What are your overall impressions of the intervention? Is there anything that could be changed about it
12. Are there particular elements that you will continue to use in the future? If there are, what would help you to keep using them? If you are not planning to keep using them, can you tell me why this is the case?

Site #9 interview questions for clients, 2-4 weeks after using teach-back, the learning styles tool and the appointment planner with their clinicians

Purpose of the interview: To investigate clients' perspectives on the value of teach-back/ learning styles tool/ appointments planner; and to identify early outcomes and any challenges.

Intervention delivery. **Teach-back method**

1. I'd like to ask you about when your HARP worker first asked you to repeat back to them what they had just taught you. Can you remember what it was they taught you? *[can prompt them if they don't remember, and the clinician has provided that information].*
2. Can you tell me a bit more about that? What do you remember now about what they taught you?
3. How did you feel about being asked to "teach-back" to your clinician?
4. You learned to do *[insert skill].* How do you feel about managing this skill after you leave the HARP program?

Remembering appointments

5. What were some of the strategies about remembering appointments that you and your clinician discussed that you feel might work for you?
6. How did you feel about discussing ways to remember health appointments with your clinician?
7. How do you feel about using these strategies after you leave the HARP program?

Learning styles tool

8. Thinking back to when your HARP worker first asked you to think about how you like to learn new things, was that something that was easy or hard to think about? Can you remember what you said was your 'preferred' way to learn new things?
9. What did you like or not like about using the tool?

Site #9 interview questions for clinicians, 4-5 weeks after using teach-back, learning styles tool and appointment planning with clients

Purpose of the interview: To investigate clinicians' perspectives on their early involvement in the Ophelia project including delivery, early outcomes and any challenges

Context/ background

13. How would you describe the Ophelia intervention in your organisation? What were the specific elements?

Delivery of the intervention/ early evidence of effect

14. Can you give me some examples of how you have used teach-back in your practice? How frequently are you using it?
15. I'm particularly interested in finding out about strategies that are helpful with more complex clients, or challenges you have come across and ways you have got around these. Can you give me any examples of these?
16. What sort of things might have stopped you from using teach-back? Can you give me some examples of this?
17. Can you tell me about the learning styles tool. How have you been using that? Can you give me some examples?
18. If you have used it, how did you adapt your teaching approach to meet your client's preferred learning style? Any examples?
19. What sort of feedback have you had from clients who used the learning styles tool?
20. Can you give me some examples of how and when clients have used the appointment planner?
21. How many clients used the appointment planner? What sort of feedback have you had from clients who used it?

Lessons learned

22. What are your overall impressions of the intervention? Is there anything that could be changed about it
23. Are there particular elements that you will continue to use in the future? If there are, what would help you to keep using them? If you are not planning to keep using them, can you tell me why this is the case?
